# Evaluation of an improved computer-aided detection system for Barrett’s neoplasia in real-world imaging conditions

**DOI:** 10.1055/a-2642-7584

**Published:** 2025-08-19

**Authors:** Martijn R. Jong, Rixta A. H. van Eijck van Heslinga, Carolus H. J. Kusters, Tim J. M. Jaspers, Tim G. W. Boers, Lucas C. Duits, Roos E. Pouw, Bas L. A. M. Weusten, Alaa Alkhalaf, Fons van der Sommen, Peter H. N. de With, Albert J. de Groof, Jacques J. Bergman, on behalf of the BONSAI Consortium

**Affiliations:** 1 Department of Gastroenterology and Hepatology, Amsterdam Gastroenterology, Endocrinology and Metabolism, Amsterdam UMC, University of Amsterdam, Amsterdam, The Netherlands; 2 Department of Electrical Engineering, Eindhoven University of Technology, Eindhoven, The Netherlands; 3 Department of Gastroenterology and Hepatology, St Antonius Hospital, Nieuwegein, The Netherlands; 4 Department of Gastroenterology and Hepatology, University Medical Center Utrecht, Utrecht University, Utrecht, The Netherlands; 5 Department of Gastroenterology and Hepatology, Isala Hospital, Zwolle, The Netherlands

## Abstract

**Background** Computer-aided detection (CADe) systems may improve detection of Barrett’s neoplasia. Most CADe systems are developed with data from expert centers, unrepresentative of heterogeneous imaging conditions in community hospitals, and therefore may underperform in routine practice. We aimed to develop a robust CADe system (CADe 2.0) and compare its performance to a previously published system (CADe 1.0) under heterogeneous imaging conditions representative of real-world clinical practice.

**Method** CADe 2.0 was improved through a larger and more diverse training dataset, optimized pretraining, data augmentation, ground truth use, and architectural adjustments. CADe systems were evaluated using three prospective test sets. Test set 1 comprised 428 Barrett’s videos (114 patients across five referral centers). Test set 2 addressed endoscopist-dependent variation (e. g. mucosal cleaning and esophageal expansion), with paired subsets of high, moderate, and low quality images (122 patients). Test set 3 addressed endoscopist-independent variation, with 16 paired subsets of 396 images (122 patients), each being based on a different software image-enhancement setting.

**Results** CADe 2.0 outperformed CADe 1.0 on all three test sets. In test set 1, sensitivity increased significantly from 87 % to 96 % ( *P* = 0.02), while specificity remained comparable (73 % vs. 74 %; *P* = 0.73). In test set 2, CADe 2.0 consistently surpassed CADe 1.0 across all image quality levels, with the largest performance gains observed on lower quality images (sensitivity 78 % vs. 61 %; specificity 89 % vs. 77 %; area under the curve 89 % vs. 75 %). In test set 3, CADe 2.0 showed improved performance and displayed reduced performance variability across enhancement settings.

**Conclusion** Based on several key improvements, CADe 2.0 demonstrated increased detection rates and better robustness to data heterogeneity, making it ready for clinical implementation.

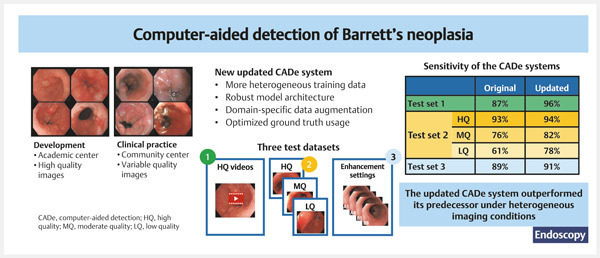

## Introduction

Barrett's esophagus (BE) patients have an increased risk of developing esophageal adenocarcinoma (EAC). Timely detection and treatment of EAC is associated with improved outcomes 1 . BE patients therefore undergo regular surveillance endoscopies 2 ; however, early neoplasia is difficult to recognize and may be missed 3 4 5 6 7 . Computer-aided detection (CADe) systems may assist endoscopists in the detection of early BE neoplasia 8 9 10 11 .

Recently, we developed a CADe system for Barrett’s neoplasia on the largest BE dataset to date and extensively evaluated this system in an ex-vivo benchmarking study 12 . The system outperformed a large group of general endoscopists for neoplasia detection and performed on a par with BE experts. More importantly, the detection rate of general endoscopists significantly increased when they received CADe assistance. However, similarly to nearly every endoscopic AI system, all training and test data in this study was acquired by expert endoscopists in academic hospitals according to standardized protocols. This resulted in high quality imaging conditions that are unrepresentative of clinical practice.

The majority of endoscopic BE surveillance is performed by general endoscopists in community centers, where the endoscopic image quality is subject to considerable variation. Such variation can be attributed to endoscopist-dependent factors (e. g. skill, experience), as well as endoscopist-independent factors (e. g. endoscopy equipment, specific endoscopic software settings). This results in a so-called “domain gap,” a phenomenon where an AI system underperforms because of a mismatch between the dataset used for its development and the data on which it is deployed 13 14 15 . We have previously shown that endoscopic AI systems are vulnerable to these domain gaps 16 17 18 . Ensuring *robustness* to data heterogeneity is therefore crucial for successful implementation of AI systems in clinical practice.

In this study, we aimed to develop a more robust CADe system for the detection of Barrett’s neoplasia, from now on referred to as CADe 2.0, designed for use in routine clinical practice. We evaluated its performance under heterogeneous imaging conditions representative of real-world clinical practice and compared it to its predecessor, from now on referred to as CADe 1.0.

## Methods

### Study design

In this study, we first integrated multiple improvements into our CADe 2.0 system in order to enhance its performance and robustness to data heterogeneity. These improvements included self-supervised pretraining, increasing the quantity and diversity of the training data, model-architecture optimization, specific data augmentation methods, and optimized usage of ground truth segmentations. We then compared its performance to CADe 1.0 for the detection of Barrett’s neoplasia using three prospectively collected, independent test sets. These test sets aimed to reflect the image diversity encountered in daily endoscopic practice.

The study adhered to the QUAIDE reporting guidelines for preclinical studies in diagnostic endoscopy 19 . A checklist is provided in **Table 1 s** , see online-only Supplementary materials.

### Data collection

Both CADe systems have been developed by the BONS-AI consortium (Barrett’s OesophaguS imaging for Artificial Intelligence). We collected white-light endoscopy images from consecutively enrolled, treatment-naïve BE patients undergoing regular endoscopic surveillance, or endoscopic treatment of a lesion in the Barrett segment. Patients with a visible lesion and histologically confirmed neoplasia, as well as patients without a visible lesion and no histologic evidence of neoplasia, were included. Images were excluded if substantial non-neoplastic esophageal changes were present (e. g. ulceration, scarring, or severe esophagitis).

All data were collected in a strictly anonymized manner and either originated from previous studies 10 12 or were newly acquired for this study. The protocols were identical. The participating BONS-AI centers collected prospective images using a standardized workflow for image and video acquisition, which has been described previously 10 .

### CADe 1.0 system

The CADe 1.0 system was pretrained on GastroNet-5 M, using a semi-supervised learning method 20 . GastroNet-5 M is a largely unlabeled dataset comprising over 5 million endoscopic images from the complete gastrointestinal tract 21 . The system was subsequently trained and internally validated on a dataset that consisted of both retrospectively and prospectively collected BE images. The training data comprised exclusively high quality images acquired by expert endoscopists from 15 international tertiary hospitals. It included 6337 neoplastic images and 7695 nondysplastic images originating from 1362 and 1139 BE patients, respectively.

All images used in this study were obtained with X1 and 190-series gastroscopes and processors from Olympus (Tokyo, Japan) using high definition white-light endoscopy *.* Data were captured using Medicapture USB300 and Sony HVO-4000MT recorders. The CADe 1.0 system was constructed using an EfficientNet-Lite1 encoder and a MobileNetV2 DeepLabV3 + decoder. A comprehensive description of the development of CADe 1.0 has been published elsewhere 12 .

### CADe 2.0 system

The CADe system underwent six substantial improvements. These aimed to increase the performance and robustness of the CADe system against endoscopist-dependent and endoscopist-independent image quality variation.

Table 1 summarizes the improvements implemented in CADe 2.0. Further technical details are provided in **Appendix 1 s** .

**Table 1:** Overview of improvements integrated into the CADe 2.0 system.

Aspect	CADe 1.0	CADe 2.0
Pretraining		
Dataset	GastroNet-5 M	GastroNet-5 M
Method	Semi-supervised	Self-supervised
Training data		
Patients	± 2500	± 2900
Images	± 14 000	± 39 000
Segmentations	± 2800	± 4300
Diversity	High quality	Diverse quality
Model architecture		
Architecture	CNN	Hybrid CNN-ViT
Model size	5.2 Mb	116.2 Mb
Parameters	4.6 million	29.7 million
Data augmentation	Regular augmentations	Regular augmentations + image enhancement-based augmentations
Ground truth use	Single consensus mask	Multiple consensus masks

CNN, convolutional neural network; ViT, vision transformer.

#### Pretraining method

Before training on application-specific data (e. g. BE images), computer vision algorithms are often pretrained on large publicly available datasets such as ImageNet 22 . These datasets include generic images (e. g. animals, vehicles, buildings), helping the algorithm recognize basic image features such as shapes, edges, and colors. This approach preserves valuable application-specific data for more advanced tasks (e. g. detecting Barrett’s neoplasia). Recent studies suggest that pretraining with large domain-specific datasets (e. g. general endoscopic images) instead of generic images can lead to even better results 23 24 . For this purpose, our group recently presented the GastroNet-5 M dataset 25 . GastroNet-5 M comprises roughly 5 million general endoscopic images of 500 000 endoscopy procedures and is largely unlabeled. Based on an extensive experimental framework including validation on nine separate endoscopic AI applications (e. g. colorectal polyp diagnosis and gastric cancer invasion-depth prediction) and 17 datasets, we found that pretraining using GastroNet-5 M resulted in higher diagnostic accuracy, required less application-specific training data, and led to more robust performance against data heterogeneity 25 .

For the development of CADe 2.0, we adopted a fully self-supervised learning approach called DINO 26 . DINO, like most self-supervised methods, involves learning meaningful representations of unlabeled data by making predictions on one part of an image based on another part (i. e. *pretext* tasks). Compared with the semi-supervised learning strategy, DINO performs significantly better on well-established benchmarks 26 .

#### Dataset size (patients)

The training set of CADe 2.0 was comparable to CADe 1.0 in terms of the number of patients. CADe 2.0 contained data from 1402 neoplastic and 1201 nondysplastic patients, compared with 1296 neoplastic and 1095 nondysplastic patients for CADe 1.0. In contrast, the internal validation set of CADe 2.0 was substantially larger. It included data from 97 neoplastic and 197 nondysplastic patients, compared with CADe 1.0, which had data from only 58 neoplastic and 36 nondysplastic patients. This larger internal validation set allowed for more informed developmental decisions during the training phase.

#### Dataset diversity (images/frames)

CADe 1.0 was exclusively trained and evaluated on high quality, expert-acquired images, which may lead to decreased performance on more diverse quality data 16 17 . For CADe 2.0, we introduced greater data heterogeneity by incorporating video data alongside high quality still images. Videos, even when acquired by experts, inherently capture a broader range of image quality conditions, including quality artifacts, such as a partial blurred lens or suboptimal mucosal cleaning. In our earlier studies, adding video frames to a homogeneous training dataset of high quality still images resulted in significantly more robust performance to data heterogeneity 27 . We therefore extracted an additional 6725 neoplastic and 9729 nondysplastic video frames originating from specific video sequences of 162 and 188 patients. Frames were automatically sampled using a custom-designed algorithm, which ensures a wide variety of content and image quality ( **Appendix 2 s** ). The internal validation set was similarly enriched with diverse quality video frames. An overview of the CADe 1.0 and CADe 2.0 training and internal validation set is given in **Table 2 s** .

#### Model architecture

CADe 1.0 was designed to operate within the computational constraints of a typical endoscopy processor. Therefore, a computationally efficient convolutional neural network (CNN) was selected (i. e. a quantized int8-based EfficientNet-lite1 architecture paired with a MobileNetV2-DeepLabV3 + decoder). In contrast, CADe 2.0 was not subjected to such computational limitations and employed a more advanced architecture with a CaFormer-S18 encoder 28 29 , a hybrid model that integrates both convolutional layers and transformer-based self-attention mechanisms, alongside a DeepLabV3 + decoder. This transition from a purely CNN-based model to a hybrid architecture was made deliberately, supported by recent developments in model architectures in general computer vision and findings from our group, which empirically demonstrated that transformer-based models may offer improved performance and robustness for endoscopic image analysis 30 .

#### Targeted data augmentation

**Fig. 1:**
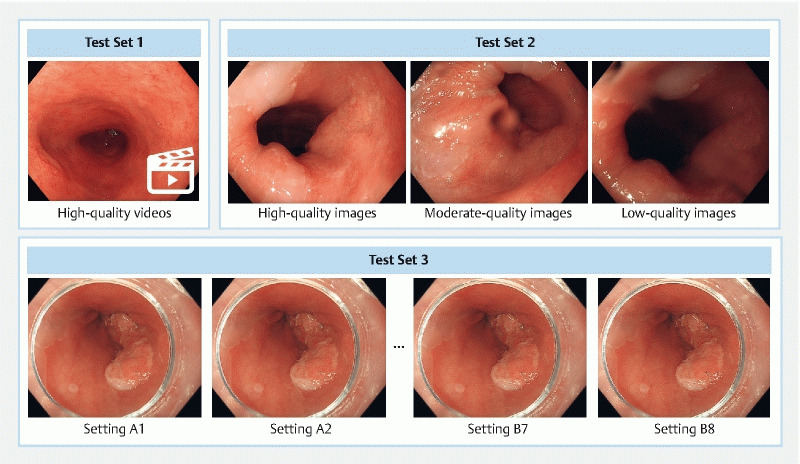
Example images from the three test sets used to evaluate the CADe systems. Test set 1 featured high quality videos recorded by expert endoscopists to assess performance under ideal imaging conditions. Test set 2 included matched video frames from the same patient, showing variations in endoscopist-dependent image quality. Test set 3 presented images displayed with different post-processing enhancement settings to evaluate robustness to endoscopist-independent image quality.

Current endoscopy systems offer a broad range of *post-processing enhancement settings* ( **Fig. 1 s** ) *.* These settings improve perceived image quality by enhancing image characteristics such as contrast, color, and texture. These settings can be adjusted based on endoscopist preferences or preinstalled by the technical services department, often resulting in significant differences between endoscopy units. In previous work we showed that the performance of endoscopic AI systems can vary substantially depending on the post-processing enhancement settings of the endoscopy system 18 31 . We also showed that, by using all available enhancement settings to augment our training data, performance variability significantly decreased.

For CADe 1.0, we only used generic data augmentation techniques (e. g. flipping, rotation, and random alteration of color and contrast). For CADe 2.0, we included additional domain-specific data augmentation by including the complete spectrum of available enhancement settings of Olympus processors into the training set. This was facilitated by a proprietary software tool capable of accurately simulating different enhancement settings, as detailed in the original publication 18 .

#### Segmentation ground truth use

Early BE lesions and particularly their outer margins are often subtle. Assessment of the outer margins of early BE lesions on images obtained with white-light endoscopy in overview may therefore be challenging. This leads to substantial interobserver variability in ground truth segmentations. For CADe 2.0, each neoplastic lesion was independently delineated by two expert endoscopists, who provided both lower likelihood and higher likelihood annotations, reflecting their graded confidence in the presence of neoplasia ( **Appendix 3 s** ). In previous studies, this approach substantially reduced variability between annotators 10 12 .

**Fig. 2:**
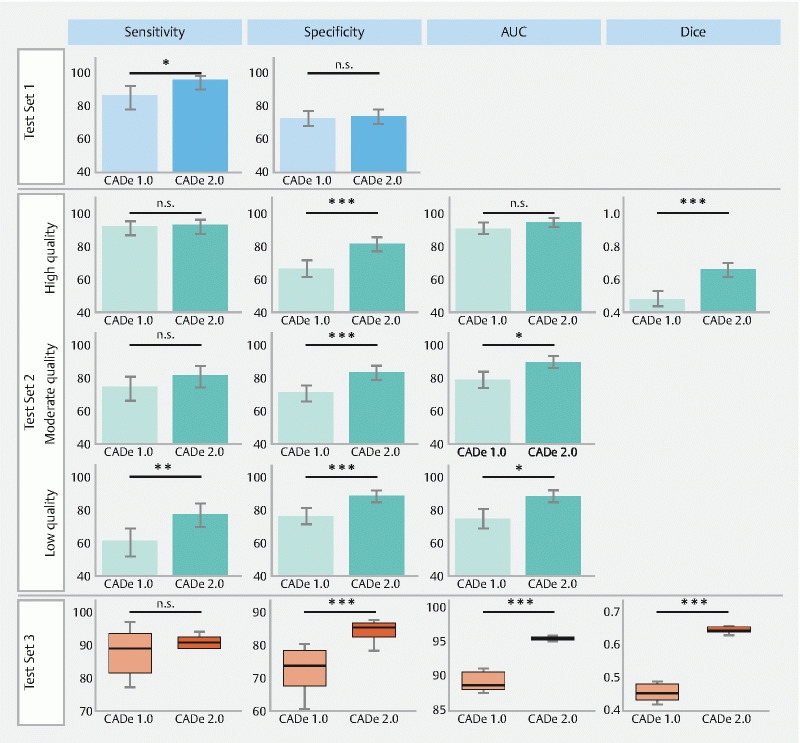
Bar graphs depicting the classification and localization performance of the CADe 1.0 and CADe 2.0 systems across the test sets, with error bars indicating confidence intervals calculated using the Wilson method. Boxplots for test set 3 illustrate classification and localization performance with differing image enhancement settings.

In the development of CADe 1.0, this nuanced information was not fully used. Instead, one single consensus segmentation was created by combining the union of both higher likelihood areas with the overlap of lower likelihood areas, resulting in a binary ground truth delineation that categorized regions as either 100 % neoplastic or 100 % nondysplastic. This approach oversimplifies clinical reality, where the transition zone of the likelihood of neoplasia is often more gradual. For CADe 2.0, we adopted a more refined approach, where varying degrees of annotator certainty were preserved during training. This method has been extensively described in a separate study 32 . An example case is depicted in **Fig. 2 s** .

### Test sets for evaluation of CAD 1.0 versus CAD 2.0

Both CADe systems were evaluated using three prospectively collected, independent test sets. These test sets are summarized in Table 2 , with example cases shown in Fig. 1 . Further details on the type of lesions in our test sets, including their macroscopic appearance and pathologic outcome, are provided in **Table 3 s** . As only lesions suitable for endoscopic resection are referred to our academic centers, our dataset consists predominantly of early stage neoplastic lesions.

**Table 2:** Overview of test sets used to evaluate the CADe 1.0 and CADe 2.0 systems.

Data set	Quality level	Total number of images 1 /videos; patients	Number of neoplastic images/videos; patients	Number of nondysplastic BE images/videos; patients
Peak performance test set		428; 114	84; 46	344; 68
Robustness to endoscopist-dependent variation test set	High	396; 122	119; 48	277; 74
	Moderate	396; 122	119; 48	277; 74
	Low	396; 122	119; 48	277; 74
Robustness to endoscopist-independent variation test set		396; 122	119; 48	277; 74

BE, Barrett’s esophagus.

1 Images in this row include both still images and video frames.

#### Test set 1: peak performance

Test set 1 comprised 428 10-second stationary videos of 114 BE patients, including 84 videos of 46 neoplastic patients and 344 videos of 68 non-dysplastic patients. Videos were collected in five BE referral centers between April 2022 and January 2024. As all videos were recorded by expert endoscopists, this test set evaluates an algorithm’s performance under ideal conditions (i. e. peak performance).

#### Test set 2: robustness to endoscopist-dependent variation

Test set 2 evaluated the CADe system's robustness to variation in endoscopist-dependent image quality. This variation includes factors such as esophageal expansion, mucosal cleaning, and the presence of blur. Test set 2 was composed of three subsets representing different levels of endoscopist-dependent image quality: high, moderate, and low. For each patient, a matched triplet of video frames was manually selected by a research fellow (R.A.H.vE.vH) and subsequently confirmed by an expert Barrett’s endoscopist (J.J.B., A.J.G.). Each triplet contained a high quality, moderate quality, and low quality frame, all captured from the same patient and at the same position within the Barrett’s segment. In total, each subset comprised 119 neoplastic and 277 nondysplastic video frames, derived from 48 and 74 patients, respectively.

#### Test set 3: robustness to endoscopist-independent variation

The third test set evaluated the CADe system's robustness to variation in endoscopist-independent image quality. Modern endoscopy systems offer a range of post-processing enhancement settings designed to improve the perceived image quality, such as contrast and color patterns. These settings can vary significantly between hospitals, often without the awareness of endoscopists. In this study, we focused on two enhancement setting types (A and B) available in current Olympus processors. Each processor can display an image using either setting A or B, each with intensity levels ranging from 1 to 8. Both settings enhance image sharpness and contrast, but differ in their specific adjustments.

This test set was derived from the high quality subset of test set 2. The subset was duplicated for every available enhancement setting (A1–A8 and B1–B8) by the use of a proprietary software tool, creating 16 subsets in total. This tool was specifically developed to be able to exactly replicate original endoscopic images with different enhancement settings 18 .

### Ground truth development

For both training and test data, the ground truth for classification of images was based on expert assessment and the corresponding histopathologic outcome. Images were labeled neoplastic if there was a visible lesion and the endoscopic resection specimen revealed high grade dysplasia or adenocarcinoma, or as nondysplastic BE if there was no visible lesion and all random biopsies were negative for neoplasia.

To provide a ground truth for lesion segmentation, neoplastic images were delineated by 16 expert endoscopists using proprietary software (Meducati, Göteborg, Sweden). All experts had both a scientific track record (i. e. had authored > 10 peer-reviewed studies) and a clinical track record (i. e. had been working for > 5 years in a tertiary referral center) in the diagnosis and treatment of Barrett’s neoplasia. Each image was delineated by at least two experts as described in the “Segmentation ground truth use” paragraph and in **Fig. 2 s** .

### Performance metrics

#### Classification performance

Classification was considered correct when the CADe system correctly identified an image or video as either neoplastic or nondysplastic BE. For videos, classification as neoplastic required the system to classify all frames as neoplastic for a time interval of at least 1 second.

#### Localization performance

Localization performance was assessed for correctly classified neoplastic images. Scores were calculated using the Dice similarity coefficient, which measures the overlap between the CADe system’s segmentation and the consensus segmentation of two expert endoscopists. Localization assessment was only performed on high quality images, as ground truth segmentation on lower quality images is inherently unreliable.

### Outcome measures

#### Peak performance (test set 1)

A comparison of the classification performance of the CADe 1.0 and CADe 2.0 systems in terms of their sensitivity and specificity was performed on test set 1.

#### Robustness to endoscopist-dependent variation (test set 2)

A comparison of the classification performance of the CADe 1.0 and CADe 2.0 systems across high, moderate, and low quality images, reporting sensitivity, specificity, and area under the curve (AUC), was performed on test set 2. Localization (Dice) scores were calculated for the high quality subset.

#### Robustness to endoscopist-independent variation (test set 3)

A comparison of the classification performance of the CADe 1.0 and CADe 2.0 systems across different enhancement settings, in terms of their median sensitivity, specificity, and AUC scores, was performed on test set 3. Localization performance was assessed in terms of the median Dice scores.

**Statistical analysis**

For descriptive statistics, categorical data are presented as frequencies and percentages. Continuous data are presented as mean (SD) or median with interquartile range (IQR) for normally distributed and skewed data, respectively. Sensitivity was defined as the proportion of all patients with a visible lesion and confirmed neoplastic pathology correctly identified as neoplastic by the CADe system. Specificity was defined as the proportion of all patients without a visible lesion and confirmed non-neoplastic pathology correctly classified as non-neoplastic by the CADe system. Sensitivity and specificity are expressed in percentages with 95 %CI, using the Wilson method.

For test sets 1 and 2, McNemar tests were used to compare sensitivity and specificity scores, DeLong tests were used for AUC comparisons, and Wilcoxon signed-rank tests for pairwise comparisons of Dice scores. For test set 3, comprising 16 copies of a single test set with varying enhancement settings, Wilcoxon signed-rank tests were applied to compare performance metrics across all settings. Correction for multiple testing was not applied, as all comparisons were predefined and performed on separate test sets targeting distinct evaluation objectives.

While the study aimed to confirm findings from previous work, it remained early phase and primarily exploratory in nature, and the results should be interpreted with some caution. Statistical analysis was performed using Python 3.8 (Python Software Foundation).

## Results

### Test set 1: peak performance

When lesions were evaluated under ideal imaging conditions, CADe 1.0 correctly classified 73/84 neoplastic videos and 252/345 nondysplastic BE videos, resulting in a sensitivity and specificity of 87 % (95 %CI 78 %–93 %) and 73 % (95 %CI 68 %–77 %), respectively. CADe 2.0 correctly classified 81/84 neoplastic videos, corresponding to a significantly higher sensitivity of 96 % (95 %CI 90 %–99 %) compared with CADe 1.0 ( *P* = 0.02). For NDBE cases, CADe 2.0 correctly classified 256/345 videos, corresponding to a specificity of 74 % (95 %CI 69 %–79 %), with no significant difference compared to CADe 1.0 ( *P* = 0.73).

Given the arbitrary nature of stand-alone performance scores for CADe systems on videos, we further assessed the performance of the CADe 1.0 and CADe 2.0 systems across various detection time cutoffs (i. e. 0.5, 1, 2, 3, and 4 seconds). CADe 2.0 consistently outperformed CADe 1.0 across all of the evaluated cutoffs. More detailed results for the individual detection time intervals are provided in **Table 4 s** .

### Test set 2: robustness to endoscopist-dependent variation

Both CADe systems were subsequently tested for robustness against endoscopist-dependent image quality variation. As shown in Table 3 and Fig. 2 , performance decreased for both systems as image quality declined from the high quality to the moderate and low quality test sets.

**Table 3:** Performance of the CADe 1.0 and CADe 2.0 systems per test set.

Test set	Quality	Metric	CADe 1.0	CADe 2.0	*P* value
Peak performance test set		Sensitivity (95 % CI)	86.9 (78.1–92.5)	96.4 (90.0–98.8)	0.02
		Specificity (95 % CI)	73.0 (68.1–77.4)	74.2 (69.3–78.5)	0.73
Robustness to endoscopist-dependent variation test set	High	Sensitivity (95 % CI)	93.2 (87.2–96.5)	94.1 (88.3–97.1)	> 0.99
		Specificity (95 % CI)	67.3 (61.6–72.6)	82.4 (77.5–86.4)	< 0.001
		AUC (95 % CI)	91.7 (88.2–95.3)	95.3 (92.6–98.0)	0.34
		Dice score (95 % CI)	0.48 (0.44–0.53)	0.66 (0.62–0.71)	< 0.001
	Moderate	Sensitivity (95 % CI)	74.8 (66.3–81.7)	82.4 (74.6–88.2)	0.10
		Specificity (95 % CI)	71.5 (65.9–76.5)	84.1 (79.3–87.9)	< 0.001
		AUC (95 % CI)	79.4 (74.2–84.6)	90.3 (86.5–94.1)	0.02
	Low	Sensitivity (95 % CI)	61.3 (52.3–69.6)	78.2 (70.0–84.7)	0.002
		Specificity (95 % CI)	77.3 (72.0–81.8)	89.2 (85.0–92.3)	< 0.001
		AUC (95 % CI)	75.2 (69.6–80.8)	89.0 (85.0–93.0)	0.05
Robustness to endoscopist-independent variation test set		Sensitivity (IQR)	89.0 (81.4–93.6)	90.8 (89.1–92.4)	0.28
		Specificity (IQR)	73.7 (67.5–78.4)	85.4 (82.6–86.7)	< 0.001
		AUC (IQR)	88.6 (88.2–90.5)	95.5 (95.3–95.7)	< 0.001
		Dice (IQR)	0.45 (0.43–0.48)	0.64 (0.64–0.66)	< 0.001

IQR, interquartile range.

On the high quality test set, CADe 1.0 achieved a sensitivity of 93 % (95 %CI 87 %–97 %), specificity of 67 % (95 %CI 62 %–73 %), and an AUC of 92 % (95 %CI 88 %–95 %) *.* CADe 2.0 yielded a comparable sensitivity of 94 % (95 %CI 88 %–97 %; *P* > 0.99), with significantly increased specificity of 82 % (95 %CI 78 %–86 %; *P* < 0.001) and a numerically higher AUC of 95 % (95 %CI 93 %–98 %; *P* = 0.34).

On the moderate quality test set, CADe 1.0’s sensitivity and AUC dropped to 75 % (95 %CI 66 %–82 %) and 79 % (95 %CI 74 %–85 %), respectively, with similar specificity of 72 % (95 %CI 66 %–77 %). CADe 2.0 outperformed CADe 1.0, with sensitivity, AUC, and specificity of 82 % (95 %CI 75 %–88 %; *P* = 0.10), 90 % (95 %CI 87 %–94 %; *P* = 0.02), and 84 % (95 %CI 79 %–88 %; *P* < 0.001), respectively.

On the low quality test set, CADe 1.0’s performance further declined to a sensitivity of 61 % (95 %CI 52 %–70 %) and an AUC of 75 % (95 %CI 70 %–81 %), with an increase in specificity to 77 % (95 %CI 72 %–82 %). CADe 2.0 demonstrated significantly improved robustness, achieving 78 % (95 %CI 70 %–85 %; *P* = 0.002), 89 % (95 %CI 85 %–93 %; *P* = 0.05), and 89 % (95 %CI 85 %–92 %; *P* < 0.001), respectively.

CADe 2.0’s ability to localize neoplasia also significantly improved compared with CADe 1.0, with respective Dice scores of 0.66 (95 %CI 0.62–0.71) and 0.48 (95 %CI 0.44–0.53; *P* < 0.001) on the high quality test set.

### Test set 3: robustness to endoscopist-independent variation

When assessing the performance of the two CADe systems across all 16 enhancement settings offered by the endoscopy system in a pair-wise manner, substantial differences in performance were observed ( Fig. 2 ; Table 3 ). CADe 1.0 achieved a median sensitivity, specificity, AUC, and Dice score of 89 % (95 %CI 81 %–94 %), 74 % (95 %CI 68 %–78 %), 89 % (95 %CI 88 %–91 %), and 0.45 (95 %CI 0.43–0.48), respectively. In comparison, CADe 2.0 reached a median sensitivity, specificity, AUC, and Dice score of 91 % (95 %CI 89 %–92 %; *P* = 0.28), 85 % (95 %CI 83 %–87 %; *P* < 0.001), 96 % (95 %CI 95 %–96 %; *P* < 0.001), and 0.64 (95 %CI 0.64–0.66; *P* < 0.001). An example case illustrating the improved performance of CADe 2.0 is shown in **Fig. 3 s** .

## Discussion



**Video 1** Examples illustrating how the CADe 2.0 system is able to improve detection of subtle lesions, while also coping with lower quality images and differing enhancement settings.


Most endoscopic AI systems are currently developed in academic centers based on data acquired by expert endoscopists using standardized protocols. These imaging conditions do not however reflect the variability encountered in community practice, posing significant challenges for clinical implementation 17 18 27 .

In this study, we present multiple improvements in an updated CADe system for Barrett’s neoplasia detection (CADe 2.0). This system was specifically designed to be more robust against data heterogeneity compared with its predecessor (CADe 1.0) 12 . To evaluate these improvements, we assessed the performance of both CADe systems using three prospectively collected, independent test sets.

Our findings show that CADe 2.0 significantly outperformed CADe 1.0 in detecting Barrett’s neoplasia under ideal imaging circumstances. More importantly, CADe 2.0 exhibited enhanced robustness across a three-tiered test set simulating varying levels of endoscopist-dependent image quality and every post-processing enhancement setting offered by the latest Olympus endoscopy processor, simulating varying levels of endoscopist-independent image quality. These results highlight the potential of the CADe 2.0 system to maintain reliable performance when confronted with the diverse imaging conditions of clinical practice. Some visual examples are given in Video 1 .

First, the CADe systems were evaluated using high quality videos originating from five BE referral centers. CADe 1.0 achieved sensitivity and specificity scores of 87 % and 73 %, respectively, consistent with its originally reported performance on another test set 12 . CADe 2.0 significantly outperformed CADe 1.0, achieving sensitivity and specificity scores of 96 % and 74 %.

To account for the arbitrary nature of stand-alone video performance metrics (e. g. a detection lasting 0.9 seconds would not qualify as a detection, while one lasting 1.1 seconds would), we also assessed CADe performance across a range of different detection time cutoffs. Across all cutoffs, CADe 2.0 consistently demonstrated higher detection rates ( **Table 4 s** ). Moreover, sensitivity and specificity scores remained relatively stable across different cutoffs, highlighting the improved consistency of the CADe 2.0 system compared with CADe 1.0. For example, with a detection time cutoff of 4 seconds, the sensitivity of CADe 1.0 dropped to 54 %, whereas CADe 2.0 retained a sensitivity of 82 %.

This improved consistency also suggests the potential to bridge the performance gap between stand-alone CADe performance and the joint performance of endoscopists assisted by CADe, as reported in the original CADe 1.0 publication 12 . In that study, although endoscopists’ performance significantly improved with CADe assistance, their sensitivity lagged by over 10 % compared with the stand-alone performance of the CADe system. A possible explanation is that endoscopists may not act on very short CADe detections, while CADe systems are evaluated purely based on such arbitrary cutoffs. By providing more consistent detections, the CADe 2.0 system may address this limitation; however, this hypothesis requires formal evaluation in a benchmark study before definitive conclusions can be drawn.

Further to the evaluation under ideal circumstances in test set 1, the two CADe systems were also evaluated under more heterogeneous imaging conditions, which better reflect routine practice.

Test set 2 examined robustness to endoscopist-dependent variation using three subsets representing varying different levels of endoscopic image quality. The CADe 2.0 system outperformed CADe 1.0 in both neoplasia classification and localization on the high quality subset. Notably, as image quality decreased, the performance gap between the systems widened, further emphasizing the increased robustness of the CADe 2.0 system. For example, sensitivity for high quality images was 93 % for CADe 1.0 and 94 % for CADe 2.0, whereas for low quality images, sensitivity was 61 % and 78 %, respectively. An example case is provided in **Fig. 4 s** .

Test set 3 assessed robustness to endoscopist-independent variation using 16 paired subsets of identical images displayed using the full range of enhancement settings offered by Olympus endoscopy processors. The CADe 2.0 system not only outperformed CADe 1.0 for all performance metrics, but also demonstrated substantially greater consistency across different enhancement settings ( **Fig. 3 s** ). For instance, the median specificity (IQR) for CADe 1.0 was 10 % (68 %–78 %), compared with 4 % (83 %–87 %) for CADe 2.0. This is highly relevant as these settings, often preconfigured by technical services and unfamiliar to most endoscopists, can vary widely between hospitals or even endoscopy suites.

These findings highlight that the updated CADe 2.0 system is more robust under suboptimal imaging conditions, whether caused by endoscopist-dependent factors such as suboptimal esophageal expansion, motion blur, or inadequate cleaning, or by processor-specific enhancement settings. This improved robustness enhances the likelihood of successful clinical implementation of the CADe 2.0 system.

The observed improvements in both absolute performance and robustness of the CADe 2.0 system can be attributed to two key factors: the underlying training data and technological advancements in the system. First, the updated system benefited from a larger and more diverse training dataset. While the CADe 1.0 system was trained on a substantial dataset, it relied on a relatively small internal validation set. In contrast, the CADe 2.0 system allocated a greater proportion of new patient data for internal validation, enabling better decision-making during the system’s development.

Additionally, the training data for CADe 2.0 was more heterogeneous, encompassing a broader spectrum of video frames representing varying levels of image quality and content. In previous work, we have demonstrated that such diversity in training data improves algorithm robustness 27 . Frames were carefully selected using an algorithm designed to maximize variability in image characteristics, while adhering to predefined quality thresholds. Furthermore, the CADe 2.0 system incorporated a wide range of image enhancement settings used by current endoscopy systems. Modern endoscopy processors often include software settings that subtly alter image characteristics such as contrast, color, and sharpness to improve perceived image quality. While these changes are often imperceptible to human observers, a prior study by our group showed that such settings can significantly impact AI performance 18 . By including these variations in its training data, CADe 2.0 became more robust to differences in processor-specific settings.

The CADe 2.0 system also benefited from significant technological advancements. Its predecessor was designed to operate on a standard endoscopy processor, with limited dedicated computational resources to run an AI algorithm. The current trend in endoscopy is however the increasing availability of more powerful local computational resources, alongside the introduction of cloud-based AI systems 33 . Therefore, our new system was developed using a state-of-the-art architecture with significantly greater computational capacity. Combined with an optimized pretraining approach, this advancement greatly contributed to the improved performance and robustness.

This study has several unique features. First, our CADe system was trained on the largest dataset for BE to date. The structure of the BONS-AI consortium, involving 15 BE referral centers across seven countries, facilitated extensive and heterogeneous data collection, including a large number of prospectively acquired cases and a comprehensive set of ground truth segmentations provided by 14 international Barrett’s experts. Second, this is among the first studies to address the critical issue of domain shift and its implications for AI implementation in endoscopy. Moreover, it is the first to present an endoscopic AI system specifically designed to improve robustness against the data heterogeneity encountered in routine clinical practice. Importantly, all improvements implemented to bridge the domain gap were individually evaluated in prior studies, significantly enhancing the validity of our findings 17 18 21 25 26 27 29 30 32 . Third, all test datasets were prospectively collected, each dedicated to a specific evaluation target. Robustness test sets were carefully designed to encompass a wide range of endoscopic image qualities and paired on a patient basis, ensuring reliable and reproducible results.

This study also has limitations. First, we did not individually assess each performance- or robustness-enhancing method. Consequently, it is challenging to determine which factor contributed most to the improved performance of the CADe 2.0 system; however, as previously noted, all methods have been independently evaluated in separate studies. Furthermore, our group will publish an ablation study of all technical design choices in a separate, more technically oriented journal. Second, this study focused on directly comparing the two CADe systems. Unlike previous studies by our group, all evaluations were conducted in a stand-alone setting, without a benchmarking performance of endoscopists when assisted by the CADe system. It is well established that high stand-alone AI performance does not necessarily translate to improved performance of endoscopists supported by AI 9 12 ; however, we have already demonstrated that the CADe 1.0 system significantly improved detection rates when used in collaboration with endoscopists. The updated CADe system detected more lesions, provided more stable predictions, and displayed more robust performance under various sources of image quality heterogeneity. This strongly suggests that endoscopists using the updated system will also benefit from its improvements.

Third, although substantially more diverse, all data in this study still originated from expert centers. Ideally, algorithms should be trained and tested using datasets that include images from nonexpert centers; however, several practical limitations, primarily the low prevalence of Barrett’s neoplasia in community hospitals, make this approach practically unfeasible 34 . Fourth, while this study addressed several relevant sources of data heterogeneity encountered in clinical practice, other potential factors remain unexplored. For instance, all data in this study were captured using Olympus equipment, while variations between endoscope manufacturers and equipment series is likely to similarly impact generalizability. Finally, even robust AI systems depend on a certain level of endoscopic quality. Lesions obscured by mucus or blind spots can still lead to missed diagnoses, so maintaining high procedural standards is therefore critical. Computer-aided quality (CAQ) systems could play a key role in supporting endoscopists here and should be investigated in future studies 35 .

In conclusion, we present an updated CADe system for early Barrett’s neoplasia detection, aimed at bridging the domain gap between performance in expert centers and community-based centers. The system demonstrated significant performance improvement compared with its predecessor. More importantly, this CADe system also displayed better robustness against real-world image quality variation, which is crucial for successful implementation into clinical practice. With the advancements of this robust algorithm, we are currently evaluating its real-time performance in clinical practice within a multicenter prospective study.

## Data availability

Results can be shared upon reasonable request by contacting m.jong3@amsterdamumc.nl .

## Supplementary Material

Supplementary material Zusatzmaterial
