# Supplementary material for: Evaluation of an improved computer-aided detection system for Barrett’s neoplasia in real-world imaging conditions
Source: Endoscopy. 2025 Aug 19;57(12):1327–37. doi: 10.1055/a-2642-7584 (PMC12659990; doi:10.1055/a-2642-7584)
Supplement: Supplementary file 2 — Supplementary material [file 25073supmat_10-1055-a-2642-7584.pdf]

SUPPLEMENTARY MATERIAL

Evaluation of an improved computer-aided detection system for Barrett's neoplasia in real-world imaging conditions

M.R. Jong*, R.A.H. van Eijck van Heslinga*, C.H.J. Kusters, T.J.M. Jaspers, T.G.W. Boers, L.C. Duits, R.E. Pouw, B.L.A.M. Weusten, A. Alkhalaf, F. van der Sommen, P.H.N. de With, A.J. de Groof, J.J. Bergman on behalf of the BONSAI Consortium
* Joint first authors

CONTENTS

	Page
Members and collaborators of the BONSAI-consortium	2
Table 1s: QUAIDE checklist	3
Appendix 1s: CADe 2.0 algorithm development	4
Appendix 2s: Video frame sampling method	4
Table 2s: Overview of data sets used for development of CADe 1.0 and CADe 2.0	5
Figure 1s: Full range of enhancement settings of the EXERA III system (Olympus, Tokyo)	5
Appendix 3s: Delineation process and contributing experts	6
Figure 2s: Example of the delineation process used to create ground truth segmentations	7
Table 3s: Description of the Peak performance test set	8
Table 4s: Performance of CADe 1.0 and CADe 2.0 across different detection time cut-offs	8
Figure 3s: Performance variability across different enhancement settings	9
Figure 4s: Predictions on high, moderate and low-quality images	10

Endoscopy | DOI 10.1055/a-2642-7584 | © The Author(s).

MEMBERS AND COLLABORATORS OF THE BONSAI-CONSORTIUM

The authors would like to thank the members and collaborators of the BONS-AI Consortium, whose contributions are acknowledged below in alphabetical order:

- Alaa Alkhalaf, Isala Hospital, Zwolle, the Netherlands;
- Lorenza Alvarez Herrero, St. Antonius Hospital, Utrecht, the Netherlands;
- Francisco Baldaque-Silva, Karolinska University Hospital, Stockholm, Sweden;
- Maximilien Barret, Cochin Hospital, Paris, France;
- Jacques J Bergman, Amsterdam UMC, Amsterdam, the Netherlands;
- Torsten Beyna, Evangelisches Krankenhaus Düsseldorf, Düsseldorf, Germany;
- Tim G Boers, TU Eindhoven, Eindhoven, the Netherlands;
- Lucas C Duits, Amsterdam UMC, Amsterdam, the Netherlands;
- Rixta van Eijck van Heslinga, Amsterdam UMC, Amsterdam, the Netherlands;
- Peter Elbe, Karolinska University Hospital & Karolinska Institute, Stockholm, Sweden;
- Albert J de Groof, Amsterdam UMC, Amsterdam, the Netherlands;
- Martin H Houben, HagaZiekenhuis, The Hague, the Netherlands;
- Tim Jaspers, TU Eindhoven, Eindhoven, the Netherlands;
- Martijn Jong, Amsterdam UMC, Amsterdam, the Netherlands;
- Jelmer B Jukema, Amsterdam UMC, Amsterdam the Netherlands;
- Carolus H Kusters, TU Eindhoven, Eindhoven, the Netherlands;
- Rosalie C Mallant-Hent, Flevoziekenhuis, Almere, the Netherlands;
- Guiomar Moral Villarejo, Nottingham University Hospitals NHS Trust, Nottingham, United Kingdom
- Wouter Nagengast, UMC Groningen, Groningen, the Netherlands;
- Jacobo Ortiz Fernández-Sordo, Nottingham University Hospitals NHS Trust, Nottingham, United Kingdom
- Oliver Pech, St. John of God Hospital, Regensburg, Germany;
- Roos E Pouw, Amsterdam UMC, Amsterdam, the Netherlands;
- Krish Ragunath, Royal Perth Hospital, Perth, Australia;
- Pieter Scholten, Onze Lieve Vrouwe Gasthuis, Amsterdam, the Netherlands;
- Stefan Seewald, Klinik Hirslanden, Zurich, Switzerland;
- Fons van der Sommen, TU Eindhoven, Eindhoven, the Netherlands;
- Jessie Westerhof, UMC Groningen, Groningen, the Netherlands;
- Bas L Weusten, UMC Utrecht & St. Antonius Hospital, Utrecht, the Netherlands;
- Peter H de With, TU Eindhoven, Eindhoven, the Netherlands;

Endoscopy | DOI 10.1055/a-2642-7584 | © The Author(s).

Table 1s QUAIDE (Quality assessment of AI preclinical studies in diagnostic endoscopy) checklist

Section and topic	Item #	Checklist item	Page where item is reported	Comments
**METHODS**				
Data acquisition	#1	Describe the entire data acquisition process in terms of setting, technology and endoscopist.	5	
	#2	Present data regarding the heterogeneity in data (in terms of quality of images / videos and complete disease spectrum) and methods to avoid selection bias.	6+7	
	#3	Report if the selection of cases for the test set was patient or lesion based.	8+9	
	#4	Provide technical information on image / video collection (endoscope, image-enhanced endoscopy, capturing devices), including image / video file type, compression standards and pre-processing methods.	5+S3	
Labeling and annotation	#5	State, define and justify the r eference standards used for AI development and testing (also known as annotation or ground truth).	8+9	
	#6	Indicate annotation details such as predetermined instructions, how many operators and their expertise.	8+9	
Outcomes	#7	Describe the outcome parameters chosen and their relation with clinically relevant endpoints.	10	
	#8	Report the diagnostic properties of the proposed AI task using generally accepted metrics (and explain the choice of the metrics). Provision of “raw” data is recommended.	10	
	#9	Report the level of experience of benchmarking endoscopists.	-	This study does not include benchmarking results of endoscopists. This is discussed in the Discussion section, p. 15.
Experimental set-up	#10	Describe the whole training-validation-test set pipeline, that should closely mimic the eventual application.	5-10	
Algorithm architecture	#11	Describe the base algorithm architecture and specific design choices.	7	
	#12	Report methods used for detection and countering of overfitting.	-	Supplementary mat p 3. In addition, the majority of the specific acquisition protocol has been described in previous studies as detailed in the Methods section.
	#13	Provide an exact technical description of the algorithm (at least in an appendix) in sufficient detail.	S3	
**RESULTS**				
Results presentation	#14	Report the diagnostic properties of the AI system (and comparators such as human benchmarking) for test sets (and for external validation set if applicable).	10+11	
	#15	Report the prevalence of the disease in the training / test / validation sets.	8+9+Table 2	
	#16	Provide examples of images / videos used in model development, as well as pictures (minimum) / videos (desirable) demonstrating how the system can be used in clinical practice.	Figure 1 + S1/3/4 + Sup. Video 1.	
**DISCUSSION**				
Discussion	#17	Summarize pre-existing evidence of the specific CAD application.	12	
	#18	Include a section on interpretation of outcomes, implications to practice, strengths, limitations and possible harms of the AI system. The generalizability of results should also be discussed.	12-15	

Endoscopy | DOI 10.1055/a-2642-7584 | © The Author(s).

Appendix 1s CADE 2.0 ALGORITHM DEVELOPMENT

The data pre-processing pipeline involves multiple steps designed to prepare and augment the dataset for optimal model training. First, the central active region of raw endoscopic images is resized to 256×256 pixels. Next, pixel intensity values are normalized by subtracting the channel-wise mean and dividing by the channel-wise standard deviation, based on statistics computed from the training data. To enhance generalization and effectively increase the dataset size, a diverse array of random data augmentation techniques is applied during training. The techniques encompass a range of fundamental transformations, including horizontal and vertical flipping, rotation, and adjustments to contrast, saturation and brightness. Additionally, grayscale conversion, Gaussian blurring, random affine and sharpness transformations are applied, as well as randomly introducing artificial noise corruption. The training framework utilizes the Adam optimizer with AMS-grad, applying a weight decay of 10^{-4} and $(\beta_1, \beta_2) = (0.9, 0.999)$. A dynamic learning rate scheduler is integrated, reducing the learning rate by a factor of 10 after 10 epochs of validation loss stagnation, up to a maximum of three reductions. An early stopping mechanism halts training if the validation performance does not improve by more than 0.05% over 25 epochs. The initial learning rate is set to 10^{-6}. For the classification task, the Binary Cross-Entropy (BCE) loss function is employed. For segmentation tasks, a composite function combining BCE and Dice loss. In the multiple ground-truth strategy, where all consensus ground-truth masks are provided during training, the BCE + Dice loss is extended to compute the average loss between the predicted segmentation mask and each of the four consensus ground-truth masks. To further improve generalization, label smoothing of 0.01 is applied for both losses. Although some neoplastic images lack ground-truth segmentation masks, their inclusion significantly benefits the multi-task training process by leveraging the backpropagation of classification loss from their associated labels. Balanced class representation is ensured during training by employing random sampling, maintaining an average 50-50% class distribution in each iteration. All implementations are performed in Python using PyTorch (Lightning) frameworks and are executed on 40-GB A100 GPUs (NVIDIA Corp., CA, USA).

Appendix 2s SAMPLING METHOD

An in-house tool was developed to sample *key frames* from video sequences, automating and optimizing the selection process based on both image quality and semantic relevance. This process generates two sets of video frames: high-quality (HQ) and medium-quality (MQ), categorized according to predefined quality thresholds. For each video in the training set, up to 10 key frames are selected, while a maximum of 5 key frames are extracted for each video in the validation set.

The in-house tool comprises two core components: a quality scoring model and a key frame selection model. The quality scoring model assigns a quality score ranging from 0 to 5 to each frame, while the key frame selection model generates a latent space representation of the video frames. To identify key frames, a K-Nearest Neighbor (KNN) algorithm operates within the latent space. For each video, this algorithm clusters the frames into N groups (where N=10 for training and N=5 for validation), and selects the frames closest to the cluster centers for which the quality score meets the predefined quality thresholds. Frames are categorized as MQ if their quality score lies in the range $2 < x < 3$, and as HQ if their quality score exceeds 3. This systematic approach ensures the selection of frames with semantic variation and a specific image quality.

Endoscopy | DOI 10.1055/a-2642-7584 | © The Author(s).

Table 2s Overview of data sets used for development of CADe 1.0 and CADe 2.0.

	CADe 1.0			CADe 2.0		
**Data set**	**No. of total images*; patients**	**No. of neoplastic images; segmentations; patients**	**No. of NDBE images; patients**	**No. of total images; patients**	**No. of neoplastic images; segmentations; patients**	**No. of NDBE images; patients**
ImageNet	1,200,000	NA	NA	-	-	-
GastroNet	5,084,494; unknown	NA	NA	5,084,494; unknown	NA	NA
Training set	13,846; 2,391	6,251; 2,752; 1,296	7,595; 1,095	31,913; 2,603	13,787; 3,721; 1,402	18,126; 1,201
Internal validation set	200; 96	100; 96; 58	100; 36	6,956; 294	1,618; 543; 97	5,338; 197

NDBE, non-dysplastic Barrett's esophagus.

*Images in this row includes both still images and video frames.

Figure 1s Example of the full range of enhancement settings of the EXERA III system (Olympus, Tokyo). Both type A and type B enhance fine mucosal patterns.

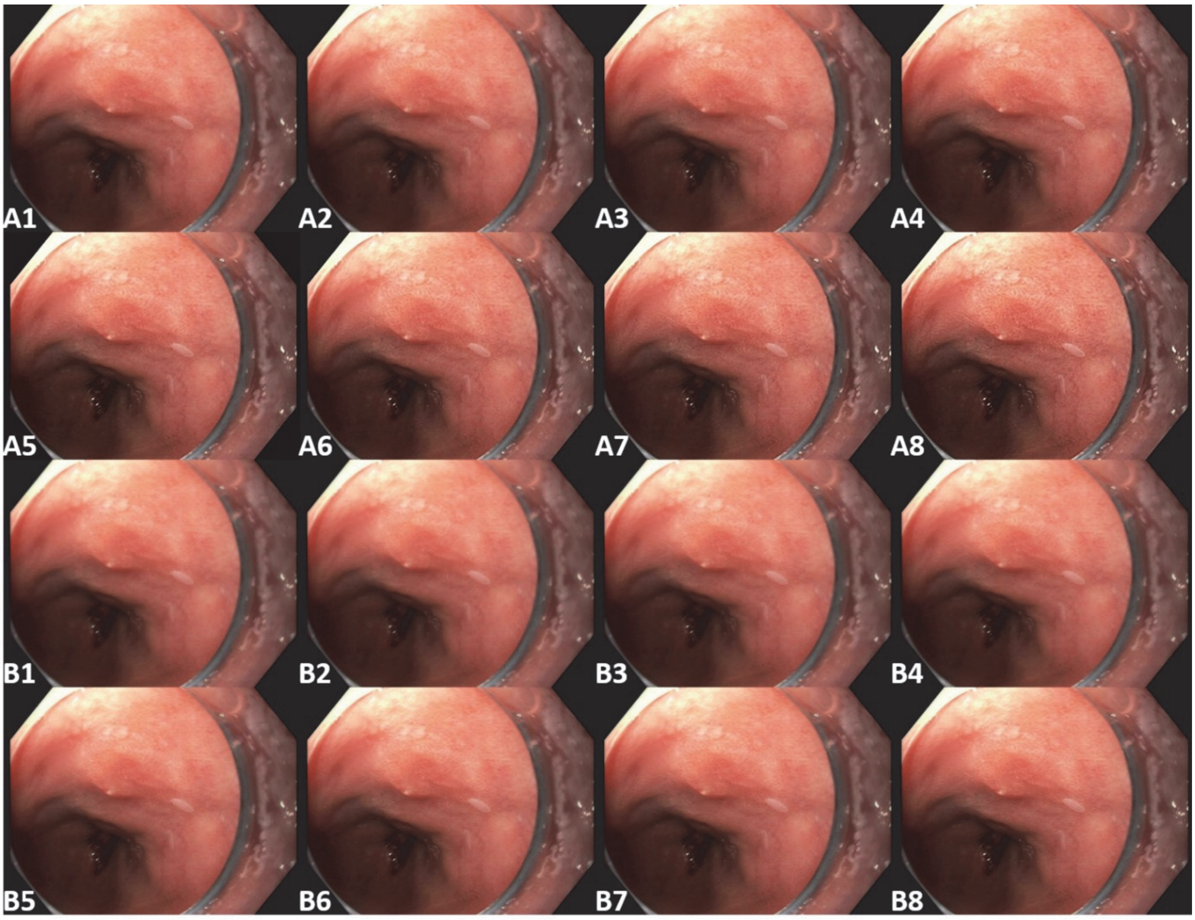

Endoscopy | DOI 10.1055/a-2642-7584 | © The Author(s).

Appendix 3s DELINEATION PROCESS AND CONTRIBUTING EXPERTS

Three research fellows reviewed all images for image quality. NDBE images with visible abnormalities and neoplasia images without visible abnormalities were excluded. A group of 16 expert endoscopists (listed below) from participating centers used an online module (Meducati AB, Göteborg, Sweden) to mark the location of the neoplastic lesion in each neoplastic image. These experts were asked to provide two delineations per image. First, they marked the outer periphery of the neoplastic lesion, including all subtle mucosal and vascular changes. Second, they only marked the area where the neoplastic lesion was most obvious, indicating the area of highest neoplasia likelihood. Subsequently, the experts were asked to score: 1) the dominant macroscopic aspect of the lesion (Paris classification 0-I, 0-II or 0-III); 2) the location of the lesion within the image (based on distance to the endoscope, angle of imaging, and whether the area was inflated or deflated); 3) the quality of the image; 4) the cleanliness of the mucosa; and 5) the subtlety of the neoplastic lesion on the image, combining all four features into a single score on a visual analog scale ranging from 1 (very subtle) to 100 (very obvious). Every neoplastic image was assessed by two experts and the overlap between their delineations was evaluated. If the overlap was insufficient (defined as a Dice score of less than 0.3), a third expert evaluated the image. The delineations made by the two experts with the highest Dice score were then used as the ground truth for neoplasia. If it was not possible to reach a consensus (Dice scores consistently lower than 0.3), the image was excluded. The ground truth delineation is defined as the combined area delineated as ‘higher likelihood for dysplasia’ by at least one expert plus the area delineated as ‘lower likelihood for dysplasia’ by both experts. A localization was defined as correct if there was any overlap between bounding box and predefined ground truth area.

The following experts contributed to delineation of neoplastic images:

- Alaa Alkhalaf, Isala Hospital, Zwolle, the Netherlands;
- Lorenza Alvarez Herrero, St. Antonius Hospital, Utrecht, the Netherlands;
- Maximilien Barret, Cochin Hospital, Paris, France;
- Jacques J Bergman, Amsterdam UMC, Amsterdam, the Netherlands;
- Torsten Beyna, Evangelisches Krankenhaus Düsseldorf, Düsseldorf, Germany;
- Lucas C Duits, Amsterdam UMC, Amsterdam, the Netherlands;
- Albert J de Groof, Amsterdam UMC, Amsterdam, the Netherlands;
- Martin H Houben, HagaZiekenhuis, The Hague, the Netherlands;
- Wouter Nagengast, UMC Groningen, Groningen, the Netherlands;
- Jacobo Ortiz Fernández-Sordo, Nottingham University Hospitals NHS Trust, Nottingham, United Kingdom
- Oliver Pech, St. John of God Hospital, Regensburg, Germany;
- Roos E Pouw, Amsterdam UMC, Amsterdam, the Netherlands;
- Krish Ragunath, Royal Perth Hospital, Perth, Australia;
- Stefan Seewald, Klinik Hirslanden, Zurich, Switzerland;
- Jessie Westerhof, UMC Groningen, Groningen, the Netherlands;
- Bas L Weusten, UMC Utrecht & St. Antonius Hospital, Utrecht, the Netherlands;

Endoscopy | DOI 10.1055/a-2642-7584 | © The Author(s).

Figure 2s Example of the delineation process used to create ground truth segmentations. (A) Original image of a Barrett's esophagus lesion. (B, C) Independent delineations by two experts, each providing lower-likelihood (lighter areas) and higher-likelihood (darker areas) annotations of neoplasia. (D) Combined annotations of both experts resulting in a gradated ground truth with varying levels of certainty. (E) Binary consensus mask used for CADe 1.0, created by combining higher-likelihood areas and overlapping lower-likelihood areas. (F) Gradual ground truth segmentation used for CADe 2.0, incorporating varying levels of annotator certainty to better reflect the subtle transitions in neoplasia likelihood.

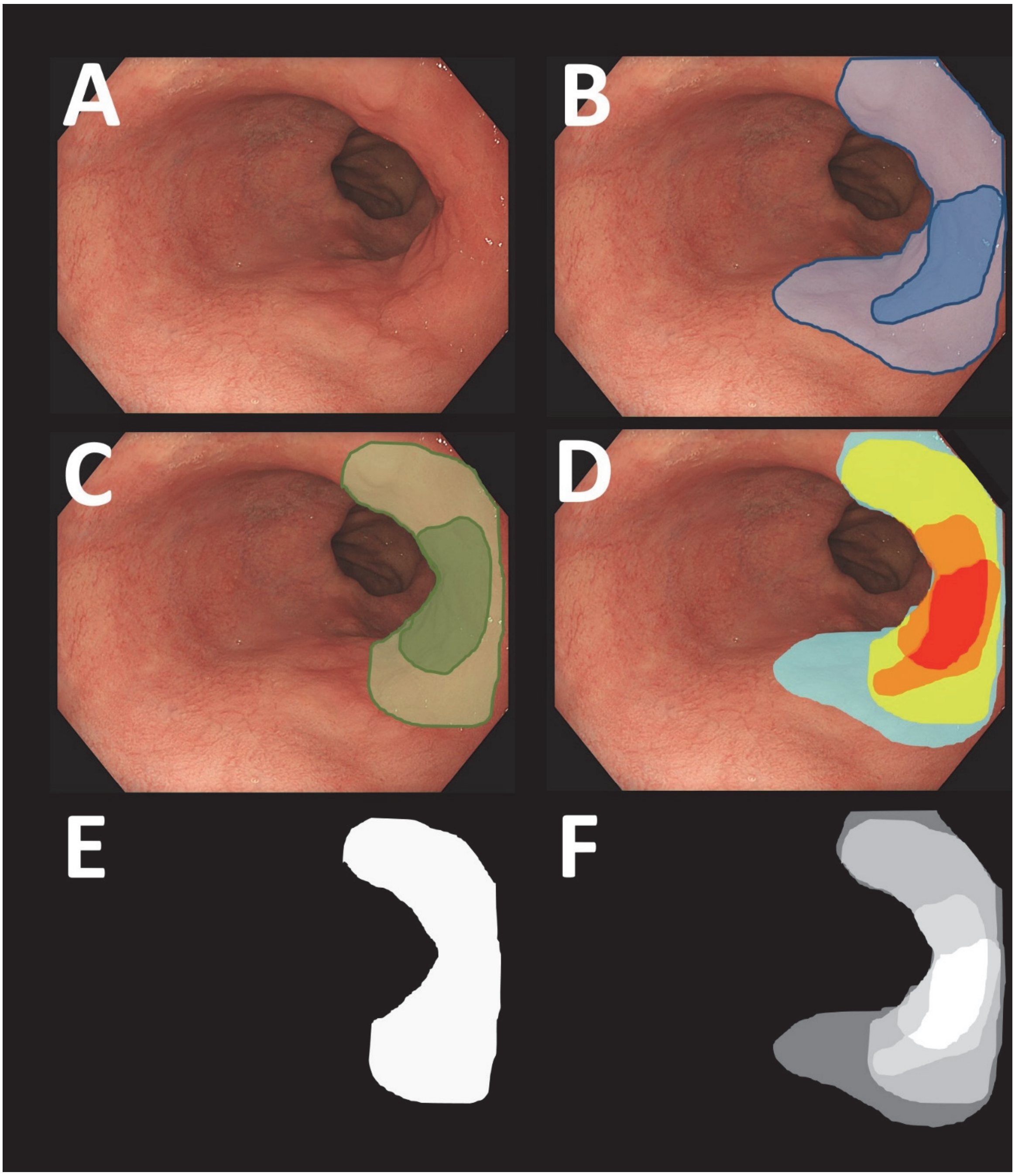

Endoscopy | DOI 10.1055/a-2642-7584 | © The Author(s).

Table 3s Description of the *Peak performance test set*. All patients included in the other two test sets (i.e. *Robustness to endoscopist-dependent variation test set* and *Robustness to endoscopist-independent variation test set)* originated from this test set.

	Peak performance test set
Patients, n (%)	
Neoplastic	46 (40.4)
Non-dysplastic	68 (59.6)
Images / Videos, n (%)	
Neoplastic	84 (19.6)
Non-dysplastic	344 (80.4)
Primary Paris type, n (%)	
0-Ip/s	2 (4.3)
0-I + 0-II	6 (13.0)
0-II	37 (80.4)
0-II + 0-III	1 (2.2)
Neoplastic pathology, n (%)	
High-grade dysplasia	15 (32.6)
Esophageal adenocarcinoma	31 (67.4)

Table 4s Performance of CADe 1.0 and CADe 2.0 on videos from the peak performance test set across different detection time cut-offs.

Cut-off (sec)	Metric	CADe 1.0	CADe 2.0	P-value
0.5	Sensitivity (95% CI)	95.2 (86.8-97.4)	97.6 (90.0-98.8)	0.625000
	Specificity (95% CI)	58.8 (53.4-63.8)	62.0 (56.7-66.9)	0.346893
1	Sensitivity (95% CI)	86.9 (76.7-91.6)	96.4 (88.4-98.1)	0.021484
	Specificity (95% CI)	73.0 (68.0-77.4)	74.2 (69.3-78.5)	0.734342
2	Sensitivity (95% CI)	72.6 (61.0-80.0)	92.9 (85.3-96.7)	0.000076
	Specificity (95% CI)	88.4 (84.6-91.3)	83.5 (79.1-87.0)	0.024092
3	Sensitivity (95% CI)	60.5 (48.8-69.4)	81.7 (71.3-87.9)	0.000040
	Specificity (95% CI)	92.8 (89.5-95.0)	89.0 (85.2-91.8)	0.053251
4	Sensitivity (95% CI)	53.8 (43.0-63.8)	82.3 (72.6-88.9)	0.000002
	Specificity (95% CI)	96.2 (93.3-97.6)	92.8 (89.5-95.0)	0.016900

Endoscopy | DOI 10.1055/a-2642-7584 | © The Author(s).

Figure 3s Example case of performance variability of the CADe 1.0 and CADe 2.0 system across different enhancement setting offered by the Olympus X1 processor. CADe 2.0 displayed more stable predictions.

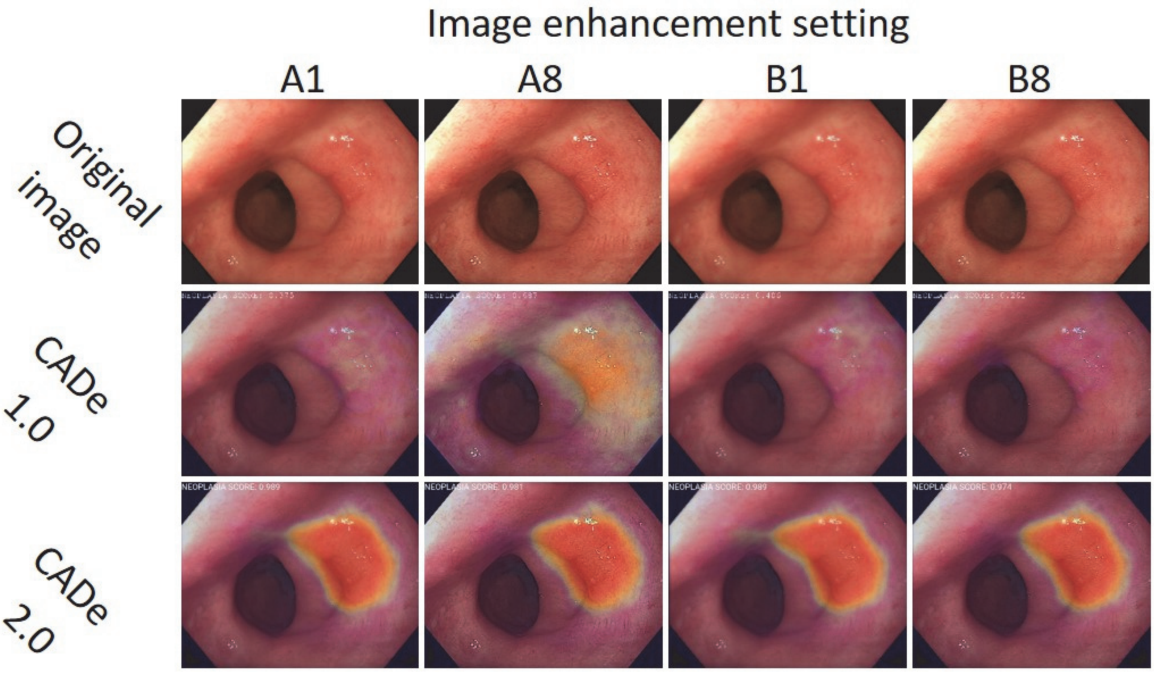

Endoscopy | DOI 10.1055/a-2642-7584 | © The Author(s).

Figure 4s Predictions of CADe 1.0 versus CADe 2.0 on high, moderate and low-quality images. CADe 2.0 demonstrates more robust predictions when confronted with lower quality input.

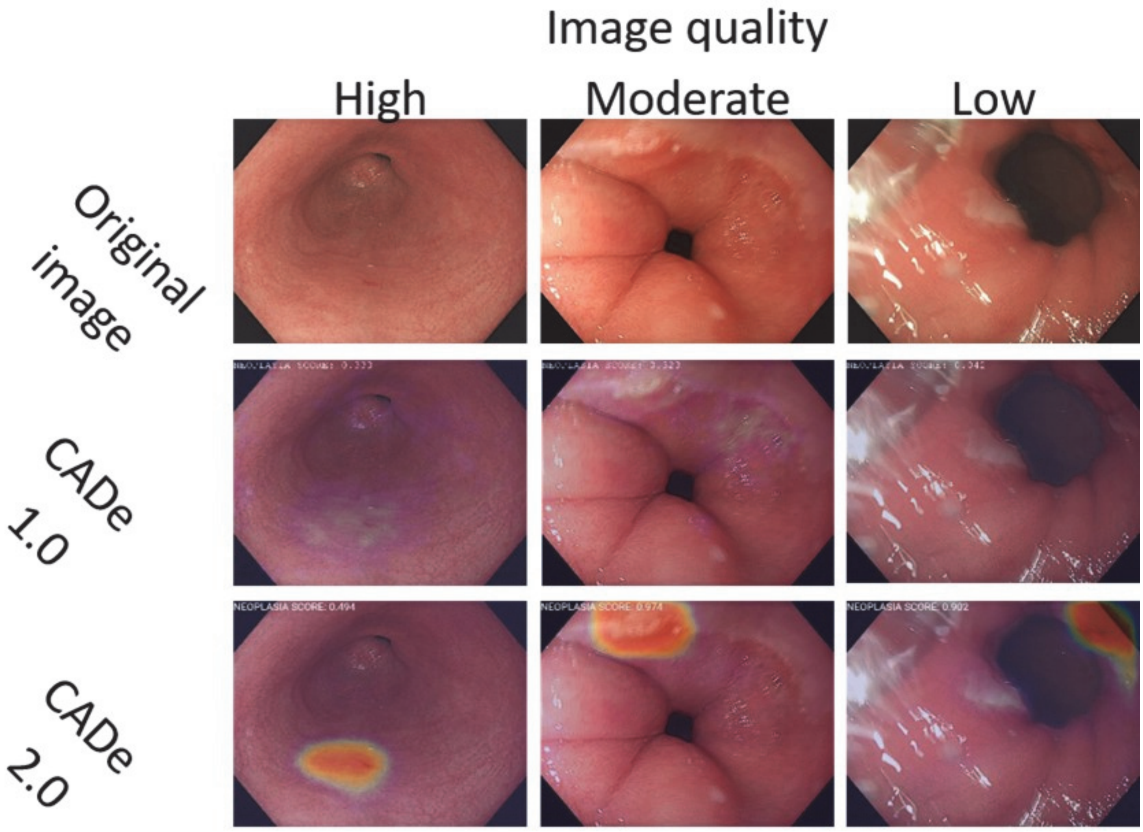

Endoscopy | DOI 10.1055/a-2642-7584 | © The Author(s).
